# Neuroanatomical Insights: Convergence and Divergence of Tinnitus with Normal or Mild Hearing Loss

**DOI:** 10.3390/biomedicines13020286

**Published:** 2025-01-24

**Authors:** Xingqian Shen, Jing Li, Hui Pan, Linlin Wang, Yangming Leng, Hongjun Xiao, Bo Liu, Wenliang Fan

**Affiliations:** 1 Department of Otorhinolaryngology-Head and Neck Surgery, ENT Institute, Union Hospital, Tongji Medical College, Huazhong University of Science and Technology, Wuhan 430022, Chinaxhjent_whxh@hust.edu.cn (H.X.); 2 Clinical Medical Research Center of Deafness and Vertigo in Hubei Province, Wuhan 430022, China; 3 Department of Radiology, Union Hospital, Tongji Medical College, Huazhong University of Science and Technology, Wuhan 430022, China; 4 Hubei Province Key Laboratory of Molecular Imaging, Wuhan 430022, China

**Keywords:** tinnitus, mild hearing loss, surface-based morphometry, voxel-based morphometry

## Abstract

**Objectives**: To explore the neuroanatomical abnormalities in idiopathic tinnitus patients by voxel-based morphometry (VBM) and surface-based morphometry (SBM) techniques. To elucidate the central plasticity in tinnitus patients with normal or mild hearing loss from the neuroanatomical insights. **Methods**: A total of 74 patients with idiopathic tinnitus (43 with normal hearing and 31 with mild hearing loss) and 98 healthy subjects were enrolled. VBM and SBM were employed to analyze neuroimaging data and identify neuroanatomical differences. **Results**: Our analysis revealed a reduction in gray matter volume and a distinctive pattern of changes in cortical surface features in patients with idiopathic tinnitus, especially in brain regions closely related to the limbic system, such as the bilateral parahippocampal gyrus, bilateral entorhinal cortex, and insula. Tinnitus patients with mild hearing loss have more extensive gray matter volume reduction, and more complex changes in cortical surface features compared to tinnitus patients with normal hearing. In addition, we also found a significant correlation between the Self-Rating Anxiety Scale (SAS), the Self-Rating Depression Scale (SDS), and Montreal Cognitive Assessment (MoCA) scores of patients with idiopathic tinnitus and cortical characteristic parameters in the above brain regions. **Conclusions**: There are extensive neuroanatomical alterations in tinnitus patients. Mild hearing loss may aggravate the reduction of gray matter volume and change the surface characteristics of the cortex. Anxiety, depression, and cognitive impairment in patients with idiopathic tinnitus may be related to neuroanatomical alterations in specific brain regions.

## 1. Introduction

Tinnitus is the illusory perception of meaningless sounds in the absence of an acoustic stimulus. About a quarter of the adults have experienced tinnitus [1], but only 10% to 15% of individuals with persistent tinnitus present for medical evaluation [2,3]. However, most of these patients have difficulty identifying the cause and may also exhibit varying degrees of anxiety, depression, poor concentration, and sleep disturbances [4,5]. This type of subjective tinnitus with unknown etiology is generally referred to as idiopathic tinnitus or primary tinnitus, regardless of the presence of sensorineural hearing loss [6]. Unfortunately, because the pathophysiological mechanism of idiopathic tinnitus and its associated symptoms is not well understood, there is still no effective targeted treatment. At present, the scientific understanding of tinnitus and its etiology has transitioned from thinking of tinnitus as solely a peripheral auditory problem to an increasing awareness that the plasticity of the central nervous system may play a critical role in idiopathic tinnitus [7,8]. Functional or structural changes in the central nervous system may be the true cause of chronic idiopathic tinnitus.

With this change, it is significant to explore the abnormalities of central structure and function in idiopathic tinnitus patients through brain imaging technology [9,10]. In recent years, an increasing number of studies have begun to focus on the microstructure of the brain and found possible central structural abnormalities in tinnitus patients. Svobodová et al. explored the effects of age, hearing loss, and tinnitus on white matter in the auditory system [11]. Huang et al. revealed possible abnormalities in the microstructure of white matter in the amygdala of tinnitus patients [12]. Voxel-based morphometry (VBM) and Surface-based morphometry (SBM) are methods to quantify the three-dimensional brain structure into a number that is easy to analyze by morphological measurement of brain images. VBM uses image intensity to visualize gray matter volume, while SBM provides cortical surface features including cortical thickness, surface area, and folding. Studies have shown that the combination of VBM and SBM can help researchers and clinicians better understand the normal neurobiological processes of the brain and improve the accuracy of morphological change detection [13].

As we know, the severity of idiopathic tinnitus is determined by the discomfort or distress it causes. It is these combined discomforts that the clinical characteristics of idiopathic tinnitus have extremely high heterogeneity. Due to the high heterogeneity of tinnitus patients, there are a range of contradictory and varied results regarding tinnitus-related structural changes in the brain [14,15]. Many comorbidities of tinnitus can also lead to abnormalities in the structure of the cerebral cortex, such as hearing loss [16], anxiety [17], depression [18], and cognitive dysfunction [19]. Especially, since 90% of tinnitus patients are accompanied by varying degrees of hearing loss [20], in the process of analyzing the brain structure changes of tinnitus patients, the unknown hearing condition seriously damages the accuracy and rigor of the research conclusions. Although Makani et al. discussed the changes in gray matter volume in tinnitus patients with different hearing levels through meta-analysis of VBM [21], there is no original study to systematically explore the neurostructural signatures in tinnitus patients with hearing loss and lack of SBM-related evidence.

In this study, we established strict inclusion and exclusion criteria to closely resemble the clinical setting and minimize the impact of comorbidities. We specifically recruited patients with idiopathic tinnitus as their main complaint, and we excluded those with an average hearing threshold exceeding 35 dB and those exhibiting obvious psychological symptoms. The patients were grouped according to their hearing level, and the neuroanatomical alterations in patients with idiopathic tinnitus with normal hearing or mild hearing loss were analyzed by VBM and SBM. It is expected to reveal the centric plasticity of tinnitus and the mechanism of tinnitus generation. We hope this study will provide a theoretical basis for personalized central targeted therapy for tinnitus patients.

## 2. Methods

### 2.1. Subjects

We included a total of 172 subjects, 74 subjects diagnosed with idiopathic tinnitus and 98 healthy subjects recruited from Union Hospital of Tongji Medical College of Huazhong University of Science and Technology, to form the basis of our study cohort. All subjects gave informed consent to a study protocol approved by the local Ethics Committee of Union Hospital of Tongji Medical College of Huazhong University of Science and Technology.

To mimic the clinical setting and minimize the impact of comorbidities as much as possible, we adopted the following inclusion criteria: (1) Tinnitus was the first chief complaint, and the course of tinnitus was ≥6 months. (2) Tinnitus was not found to be associated with a potential cause (except for sensorineural hearing loss) or a confirmed physiological state (i.e., idiopathic tinnitus). (3) Normal hearing or only mild hearing loss, that is, better ear hearing threshold is less than 35 decibels (dB). According to the 2021 World Hearing Report [22], the hearing level of a subject is determined by the four frequency (500, 1000, 2000, and 4000 Hz) pure tone hearing threshold of better ear. The external auditory canal was unobstructed and the tympanic membrane was intact. (4) With normal understanding and expression ability. Subjects were excluded if: (1) otitis externa, acute/chronic otitis media, and other related diseases of the external ear and middle ear; (2) sudden deafness, Meniere’s disease, otosclerosis, acoustic neuroma, and other inner ear or auditory nerve diseases; (3) severe central nervous system diseases or other systemic diseases such as cardiovascular and cerebrovascular diseases; and (4) any white matter lesions more than 1 mm visible on structural MRI. According to the hearing loss classification standard proposed in the World Hearing Report [22], we divided the patients with idiopathic tinnitus into 43 subjects of normal hearing group (TNH, better ear hearing threshold < 20 dB) and 31 subjects of mild hearing loss group (THL, better ear hearing threshold 20–35 dB). Healthy control subjects (HC) are people with physical and mental health, and normal hearing, without any psychological and neurological conditions such as anxiety, depression, sleep disorders, stroke, brain tumor, and white matter lesions more than 1 mm visible on structural MRI [23].

### 2.2. Clinical Evaluation

We obtained the demographic characteristics and pure tone audiometry (PTA) results of all subjects. We asked in detail about the characteristics of the medical history and associated discomfort of tinnitus patients. All tinnitus patients were examined for tinnitus loudness matching, pitch matching (Tf = tinnitus frequency), minimum masking level, and residual inhibition to standardized characteristics of tinnitus sound. The patients also completed the Tinnitus Handicap Inventory (THI) to assess the severity of tinnitus.

In order to evaluate the associated symptoms of tinnitus, we used the Montreal Cognitive Assessment (MoCA) to assess the cognitive function of all subjects, used the Self-Rating Anxiety Scale (SAS) and the Self-Rating Depression Scale (SDS) to assess anxiety and depression, and used the Pittsburgh Sleep Quality Index (PSQI) to assess sleep disturbances.

### 2.3. Magnetic Resonance Imaging Acquisition

Imaging was performed on a 3.0 Tesla MRI scanner (Ingenia 3.0 T CX, Philips Healthcare, Best, The Netherlands). Based on the standardized protocol, subjects were positioned lying on their backs and were directed to stay still throughout the surgical procedure. Earphones were given to reduce scanner noise, and foam pads were utilized to limit head movement. High-resolution T1 structural MRI images were acquired for each participant using a 3D fast gradient echo sequence, following the scanning parameters employed in our previous studies [24,25,26] (key parameters: ‘Turbo Field Echo’, TR = 8.2 s, TE = 3.8 s, FA = 8°, matix = 256 × 256, slice thickness = 0.7 mm, slice number = 257).

In order to ensure meticulous evaluation of the images, two experienced radiologists, each with over a decade of experience, independently reviewed the MRI scans. Subjects with identified MRI abnormalities, such as otitis media, acoustic neuroma, and brain tumors, were excluded from the following analysis.

### 2.4. Image Processing

The anatomical images were processed and analyzed using the CAT12 toolbox, which is part of the Statistical Parametric Mapping (SPM12: v7771) software suite (www.fil.ion.ucl.ac.uk/spm) (accessed on 24 September 2023). This versatile toolbox features specialized pipelines for both Voxel-Based Morphometry (VBM) and Surface-Based Morphometry (SBM) modules, providing a comprehensive platform for our analyses. During the processing and analysis phases, we employed the default settings in alignment with the established standard protocol (http://www.neuro.uni-jena.de/cat12/CAT12-Manual.pdf) (accessed on 24 September 2023). The application of CAT12 is well-established and supported by extensive validation in morphometric studies related to hearing disorders [24,25]. To ensure data quality, we implemented a two-step quality assurance process. Initially, all images underwent thorough visual inspection to identify and address any potential artifacts, which was carried out prior to preprocessing. Subsequently, we performed a statistical quality control assessment to evaluate the overall image quality and consistency across subjects following segmentation. This rigorous quality assurance procedure enhanced the integrity and reliability of our analyses, laying a strong foundation for our findings.

For the analysis of voxel-based morphometry (VBM), we implemented a systematic pipeline to extract meaningful structural differences from anatomical images. Initially, we normalized the images to a standard template using the diffeomorphic anatomical registration through the exponentiated Lie algebra algorithm. This process ensured that individual brains were aligned to a common reference space. Following normalization, we corrected all T1-weighted images for bias-field inhomogeneities and subsequently segmented them into gray matter (GM), white matter (WM), and cerebrospinal fluid (CSF). To examine regional differences in gray matter volume, we utilized modulated normalized gray matter maps, which captured local variations in gray matter while retaining anatomical integrity. We then applied spatial smoothing to the modulated gray matter maps with an 8 mm full-width half maximum (FWHM) Gaussian kernel. Throughout each stage of the process, we conducted visual assessments to identify any potential segmentation or registration issues. Lastly, we calculated the total intracranial volume by integrating the GM, WM, and CSF images generated during the segmentation process.

For the SBM analysis, standard protocol parameters were strictly followed to ensure a robust and consistent methodology. Segmentation, surface estimation, data resampling, and subsequent smoothing were executed using the default settings. The resulting surface parameters included cortical thickness, a local gyrification index that measures 3D surface complexity, sulcus depth, and cortical complexity represented by the fractal dimension. In accordance with best practices, a smoothing filter with a 15 mm FWHM was applied to the thickness data, while a 20 mm FWHM was used for the folding data (sulcal depth, local gyrification index, and fractal dimension) to enhance data quality. Thorough visual inspections were conducted to confirm that the surface data was free of artifacts and homogeneous, while statistical quality control ensured the overall fidelity of the images.

### 2.5. Statistical Analysis

SPSS 25.0 software (SPSS Inc., Chicago, IL, USA) was used for clinical data processing and statistical analysis. The Shapiro-Wilk test (SW test) was used to verify the normality of the measurement data, and the Levene test was used to verify the homogeneity of variance for the data with normal distribution. The measurement data with normal distribution were summarized as $\bar{x}$ ± s, and a one-way analysis of variance or *t*-test was used. The measurement data that did not conform to the normal distribution was summarized as M (P25, P75), and a non-parametric test was used. The count data were exhibited by frequency, and the chi-square test was used.

One-way analysis of variance (ANOVA) was used to identify the regions with significant differences in each of the morphometric measures (gray matter volume with VBM and cortical thickness, sulcal depth, local gyrification index, and fractal dimension with SBM) among the groups. The results were thresholded using the CAT12 toolbox at a default cluster-forming threshold (uncorrected *p* < 0.001). Age, gender, education, SAS score, SDS score, and MoCA score as covariates in the statistical analysis. All results were labeled according to the Desikan–Killiany atlas (DK40) [27]. In addition, the Pearson correlation was performed to explore the relationship between clinical and neuroimaging characteristics in idiopathic tinnitus patients.

## 3. Results

### 3.1. Demographic and Clinical Characteristics

In this study, we enrolled 74 patients with idiopathic tinnitus, which fell into the normal hearing group (TNH, n = 43) and the mild hearing loss group (THL, n = 31). Concurrently, 98 healthy control (HC) individuals were enrolled. We summarized the demographic and clinical characteristics of these subjects in Table 1.

There are no significant differences in age (*p* = 0.567), gender (*p* = 0.316), education level (*p* = 0.865), and PTA of better (0.214) or worse (0.466) ear between HC and TNH. However, the MoCA score (*p* < 0.001) of TNH was significantly lower than that of the matched HC, and the SAS (*p* < 0.001) and PQSI (*p* < 0.001) scores were significantly higher than those of the HC. Although TNH also showed an increasing trend in SDS scores, the difference with the HC was not significant (*p* = 0.068). Compared with the TNH and HC, THL showed older age (*p* < 0.001) and higher PTA threshold (*p* < 0.001), but there is no significant difference in gender (*p* = 0.605) and education level (*p* = 0.951). The scores of SAS (*p* = 0.230), SDS (*p* = 0.086) and PQSI (*p* = 0.674) were significantly different between the two groups of patients with idiopathic tinnitus with different hearing levels. Only in terms of MOCA score (*p* = 0.007), THL was significantly lower than TNH. Similarly, TNH and THL did not show significant differences in tinnitus disease characteristics such as tinnitus sides (*p* = 0.937), duration (*p* = 0.777), and tinnitus frequency (*p* = 0.648).

### 3.2. Gray Matter Volume

**Figure 1:**
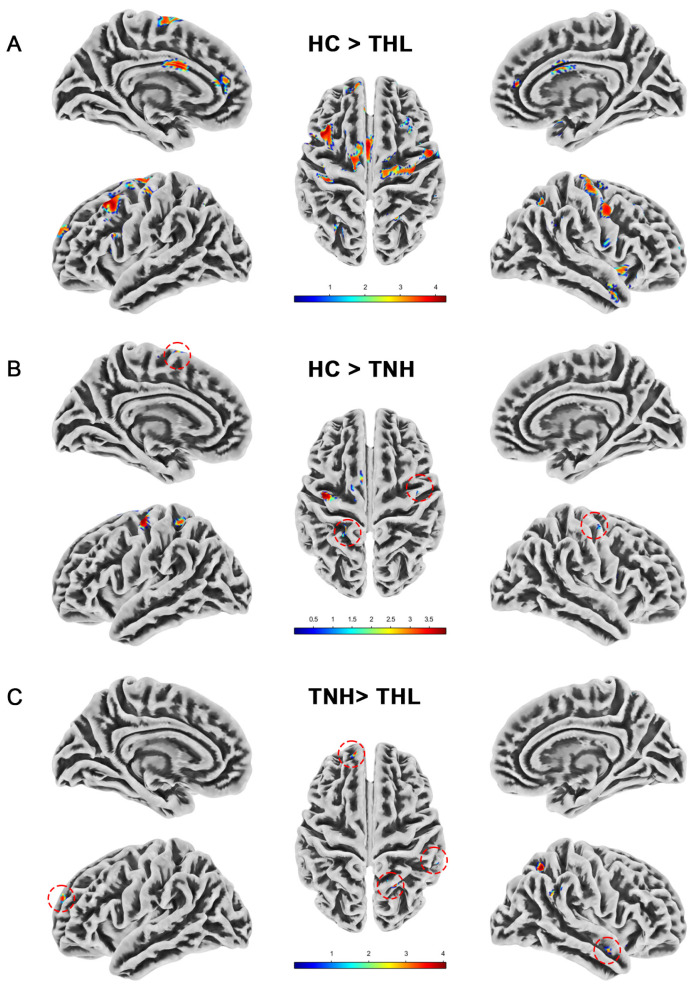
Gray matter volume differences among three groups. Red dotted circles mark brain regions that may be overlooked. (**A**) Brain areas showed significantly decreased gray matter volume in THL compared with HC. (**B**) Brain areas showed significantly decreased gray matter volume in TNH compared with HC. (**C**) Brain areas showed significantly decreased gray matter volume in THL compared with TNH. THL, idiopathic tinnitus patients with mild hearing loss; TNH: idiopathic tinnitus patients with normal hearing; HC: healthy control.

We measured the whole brain gray matter volume of all subjects by VBM and found differences in gray matter volume among the three groups. As illustrated in Table 2 and Figure 1, compared with HC, the gray matter volume of TNH was significantly reduced in the cerebrum, left precentral gyrus, and right postcentral gyrus. The reduction of gray matter volume in THL was more extensive and significant, and there were different degrees of gray matter volume reduction in the bilateral cerebral cortex and subcortex. Moreover, compared with TNH, THL has a cluster with significantly reduced gray matter volume in bilateral brain regions.

### 3.3. Cortical Thickness

**Figure 2:**
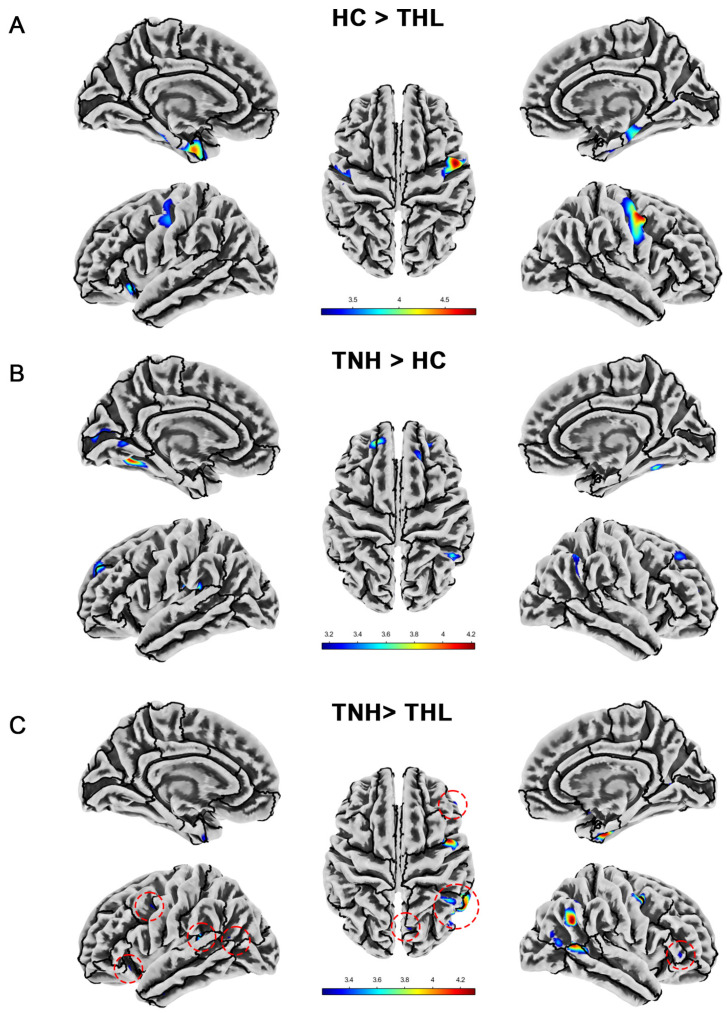
Cortical thickness differences among three groups. Red dotted circles mark brain regions that may be overlooked. (**A**) Brain areas showed significantly decreased cortical thickness in THL compared with HC. (**B**) Brain areas showed significantly increased cortical thickness in TNH compared with HC. (**C**) Brain areas showed significantly decreased cortical thickness in THL compared with TNH. THL, idiopathic tinnitus patients with mild hearing loss; TNH: idiopathic tinnitus patients with normal hearing; HC: healthy control.

As illustrated in Table 3 and Figure 2, idiopathic tinnitus patients with different hearing levels have distinctive characteristics in terms of cortical thickness alterations. Compared with HC, THL showed significant cortical thickness reduction in the bilateral parahippocampal gyrus, bilateral postcentral gyrus, bilateral entorhinal cortex, left lateral orbitofrontal cortex, left insula, and right fusiform. However, the cortical thickness of TNH in many specific regions (such as bilateral fusiform, bilateral superior frontal gyrus, left lingual gyrus, and so on) was significantly higher than that of the matched HC, rather than reduced. Similarly, compared with THL, TNH also has multiple clusters of increased cortical thickness, mainly concentrated in the right cerebral hemisphere.

### 3.4. Sulcal Depth

**Figure 3:**
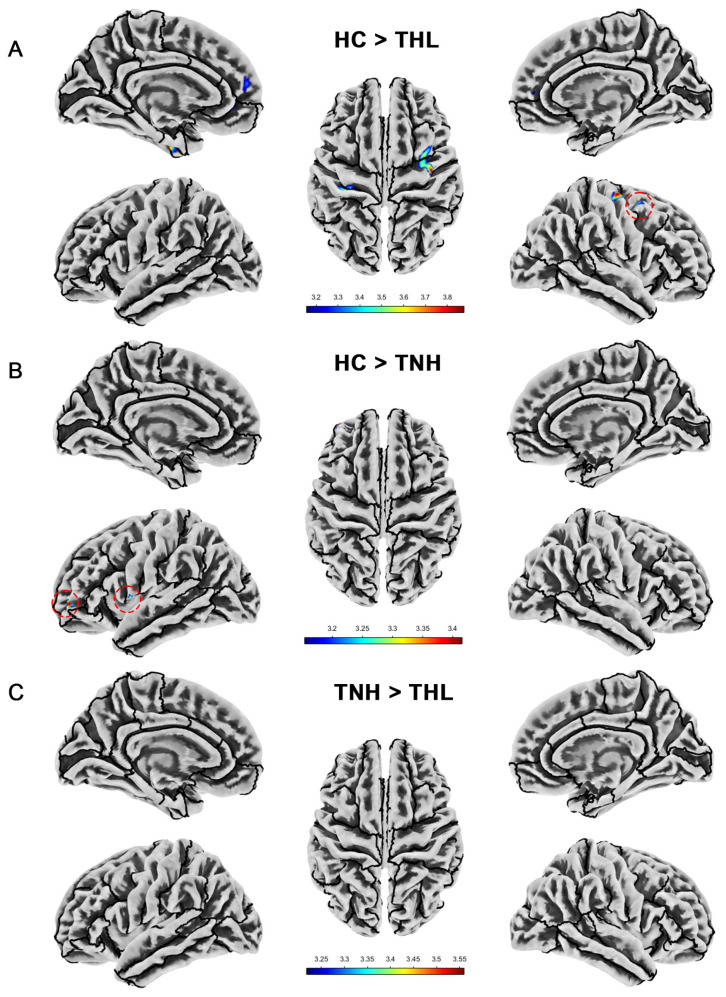
Cortical sulcal depth differences among three groups. Red dotted circles mark brain regions that may be overlooked. (**A**) Brain areas showed significantly decreased cortical sulcal depth in THL compared with HC. (**B**) Brain areas showed significantly decreased cortical sulcal depth in TNH compared with HC. (**C**) Brain areas showed significantly decreased cortical sulcal depth in THL compared with TNH. THL, idiopathic tinnitus patients with mild hearing loss; TNH: idiopathic tinnitus patients with normal hearing; HC: healthy control.

As displayed in Table 4 and Figure 3, compared with the HC group, the sulcal depth of TNH decreased in the left precentral gyrus and left rostral middle frontal cortex. THL has reduced sulcal depth in multiple clusters in the left hemisphere and one cluster in the right hemisphere. In addition, the sulcus depth of TNH in the superior temporal gyrus was deeper than that of THL.

### 3.5. Cortical Gyrification Index

**Figure 4:**
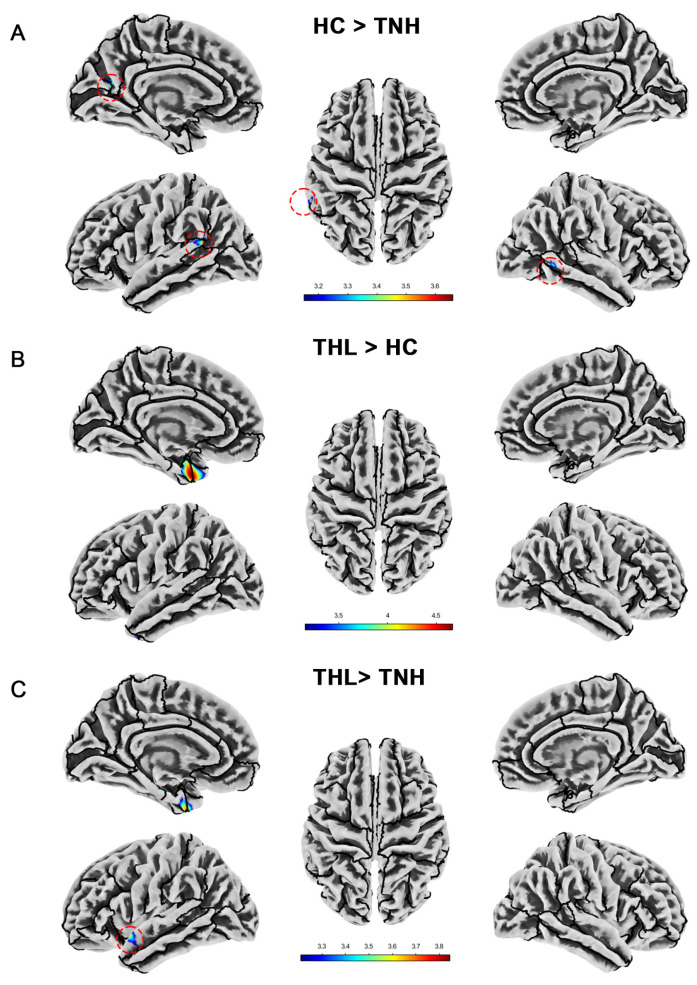
Cortical gyrification index differences among three groups. Red dotted circles mark brain regions that may be overlooked. (**A**) Brain areas showed significantly decreased cortical gyrification index in TNH compared with HC. (**B**) Brain areas showed significantly increased cortical gyrification index in THL compared with HC. (**C**) Brain areas showed significantly increased cortical gyrification index in THL compared with TNH. THL, idiopathic tinnitus patients with mild hearing loss; TNH: idiopathic tinnitus patients with normal hearing; HC: healthy control.

We show the significant differences in local cortical gyrification index among groups in Table 5 and Figure 4. The cortical gyrification index of TNH was significantly lower than that of HC in the left superior temporal gyrus, left supramarginal gyrus, left cuneus, left precuneus, left peri calcarine, and right middle temporal gyrus. However, THL did not have a decrease in cortical gyrification index in these brain regions. On the contrary, THL has a cluster with an increased cortical gyrification index at the junction of the left temporal pole and the left entorhinal cortex. Moreover, compared with TNH, the local gyrification index of THL increased in more regions of the left cerebral hemisphere.

### 3.6. Cortical Complexity

**Figure 5:**
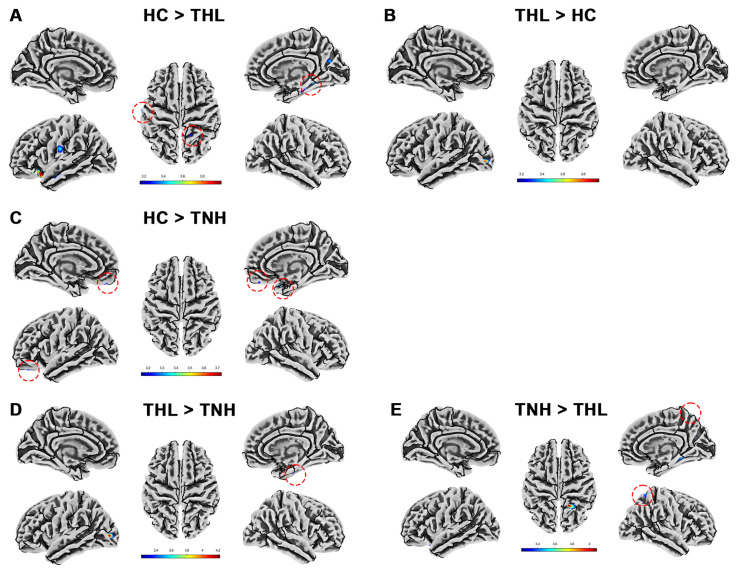
Cortical fractal dimension differences among three groups. Red dotted circles mark brain regions that may be overlooked. (**A**) Brain areas showed significantly decreased cortical fractal dimension in THL compared with HC. (**B**) Brain areas showed significantly increased cortical fractal dimension in THL compared with HC. (**C**) Brain areas showed significantly decreased cortical fractal dimension in TNH compared with HC. (**D**) Brain areas showed significantly increased cortical fractal dimension in THL compared with TNH. (**E**) Brain areas showed significantly decreased cortical fractal dimension in THL compared with TNH. THL, idiopathic tinnitus patients with mild hearing loss; TNH: idiopathic tinnitus patients with normal hearing; HC: healthy control.

Cortical complexity is quantified by the Cortical fractal dimension measured by the SBM technique. Tinnitus patients with different hearing levels showed distinct patterns of cortical complexity. We show in detail the cortical fractal dimension differences among the three groups in Table 6 and Figure 5. Tinnitus patients have clusters with reduced cortical fractal dimension in multiple specific brain regions. The fractal dimension of TNH in the left lateral occipital cortex is significantly higher than that of HC.

### 3.7. Correlation Analysis

We selected clinical characteristics such as tinnitus duration, THI scores, SAS scores, SDS scores, MOCA scores, and PQSI scores for correlation analysis with neuroimaging measures in each brain region. As revealed in Table 7, the SAS scores, SDS scores, and MoCA scores of tinnitus patients were significantly correlated with the cortical thickness, cortical gyrification index, and cortical fractal dimension of specific brain regions to varying degrees. The brain regions associated with SAS and SDS scores were highly consistent, mainly concentrated in the bilateral entorhinal cortex, fusiform, insula, and parahippocampal gyrus. Unlike the above brain regions, MoCA scores were significantly correlated with cortical thickness in the right inferior parietal lobe, left lingual gyrus, right middle temporal gyrus, bilateral precentral gyrus, and right superior temporal gyrus.

## 4. Discussion

More than 90% of tinnitus patients are accompanied by varying degrees of hearing loss. However, not all tinnitus patients seek medical help due to discomfort related to tinnitus. Patients who actively seek medical aid for tinnitus often have normal hearing or only mild hearing loss [3,6]. Early clinical practice guidelines introduced the concept of ‘Bothersome tinnitus’, which means ‘Distressed patient, affecting the quality of life and/or functional health status; the patient is seeking active therapy and management strategies to alleviate tinnitus’ [6]. Recently, some scholars have divided tinnitus into ‘Tinnitus’ and ‘Tinnitus disorder’ according to whether it is accompanied by associated suffering. ‘Tinnitus’ only describes the auditory component, but ‘Tinnitus disorder’ emphasizes its accompanying suffering [3]. In the clinical setting, it is often ‘Bothersome tinnitus’ or ‘Tinnitus disorder’ that seeks medical aid. As in this study, even idiopathic tinnitus patients with normal hearing had lower MoCA scores, higher SAS scores, and higher PQSI scores than healthy subjects matched by age, gender, education level, and hearing level. This is consistent with previous studies [28,29]. These mild scale abnormalities do not meet the diagnostic criteria of the corresponding disease, which is often directly caused by the long-term disturbance of tinnitus sounds. It is worth noting that, in addition to cognitive dysfunction, mild hearing loss, which may be due to advanced age, does not aggravate the accompanying symptoms of tinnitus, nor does it aggravate the tinnitus itself. This may be related to the fact that the patients included in this study have tinnitus as the chief complaint, and the hearing loss is mild. So, the patients have almost no social barriers caused by hearing loss; as described in the 2021 World Hearing Report, most adults have no problem conversing in quiet situations but may have problems conversing in noisy environments. Their psychological symptoms are almost entirely caused by long-term tinnitus, noise interference, and secondary sleep disorders. Mild hearing loss may have a certain impact on cognitive function, but due to the older age of THL, it cannot be determined in this study whether the decrease in MoCA score can be explained by aging factors. Although the age of THL was older in both groups of tinnitus patients, its mean age was 52.6 years, ranging from 28 to 67 years, while patients over 60 years old accounted for only 25.8% (8/31). It will be difficult to explain these mild hearing losses with age-related hearing loss. Moreover, the patients we included were chronic idiopathic tinnitus patients with a disease duration of over 6 months, who presented with tinnitus as their first complaint and often reported no hearing loss or distress caused by hearing loss. These factors collectively make it difficult to trace the cause of mild hearing loss in THL. Epidemiological investigations have shown a significant age dependency in hearing loss among tinnitus patients [30], which may also explain the older age of THL in our study.

This study quantified the neuroanatomical changes in patients with idiopathic tinnitus by morphological measurements, revealed possible neural structural abnormalities in these patients, and explored the correlation between a series of comorbid symptoms and these abnormalities in these patients. We evaluated the neuroanatomical features of these patients from multiple perspectives using gray matter volume measured by VBM and cortical thickness, sulcal depth, gyrification index, and fractal dimension measured by SBM. In each measurement, we found distinctive positive results, which may suggest that idiopathic tinnitus has a distinctive neuroanatomical alternation. The gray matter is mainly composed of neuronal cell bodies and synapses. It is mainly responsible for processing sensory input and motor output and is closely related to advanced cognitive function. We found that patients with idiopathic tinnitus have varying degrees of gray matter volume reduction, which is roughly consistent with previous studies [31,32]. However, the brain region localization does not completely overlap. Schmidt et al. found that patients with mild tinnitus (without reported hearing levels) had reduced gray matter volume in the left cingulate gyrus compared to the control group with normal hearing [31]. However, our results showed that idiopathic tinnitus patients with mild hearing loss had reduced gray matter volume in the left cingulate gyrus, while idiopathic tinnitus patients with normal hearing had no significant difference in gray matter volume in this brain region. The research results of Bianca et al. on gray matter volume in tinnitus patients mainly focus on the left parahippocampal, fusiform gyri, right inferior parietal gyrus, and precuneus. This may be due to the inclusion of subjects with clinical manifestations of anxiety and depression. Cortical thickness is a measurement of the distance between the inner and outer surfaces of the gray matter of the brain. To a certain extent, it characterizes the development and aging of the brain. Cortical thickness changes in specific regions are associated with central nervous system diseases [33]. Here, we found that tinnitus patients with mild hearing loss had reduced cortical thickness of multiple clusters including the bilateral parahippocampal gyrus compared to healthy controls. This is consistent with previous results: Changes in the hippocampus and parahippocampal gyrus are usually used as part of the ‘limbic system’ to explain the emotional response associated with tinnitus [34]. The surface complexity of the cerebral cortex was evaluated by combining the cortical sulcal depth and the gyrification index. In general, the cortical complexity of tinnitus patients showed a multi-cluster reduction, mainly in the left superior temporal gyrus, left cuneus, left precentral gyrus, and left postcentral gyrus. The cortical fractal dimension can condense the shape complexity into a single value, evaluate the changes in the local cortex of the brain as a whole, and provide quantitative evaluation [35]. The cortical complexity of tinnitus patients shows a unique complex form. For example, idiopathic tinnitus patients with mild hearing loss have reduced cortical complexity in multiple clusters of multiple brain regions, but there is a cluster with significantly increased complexity in the left lateral occipital cortex, whose main function involves object recognition.

Our results show that idiopathic tinnitus patients with mild hearing loss have extensive gray matter volume reduction in both the bilateral cerebral cortex and subcortex. This may be related to the fact that they are older [36] than the healthy control group and have mild hearing loss [37]. However, in view of the small difference in gray matter volume between the two groups of tinnitus patients, tinnitus should also be considered as one of the reasons for the reduction of extensive cortical volume. Husain et al. found that hearing loss will further aggravate the reduction of gray matter volume in patients with tinnitus, which is consistent with our results. In this study, idiopathic tinnitus patients with normal hearing were compared with matched healthy controls; the area of cortical volume reduction was mainly located in the left precentral gyrus of the left frontal lobe and cerebellum. The precentral gyrus and cerebellum are involved in multimodal sensory and sensory-motor integration, which are implicated in auditory processing. Animal experiments have confirmed that destroying the feedback loop of the paraflocculus and the auditory cortex may help maintain and reduce tinnitus [38]. However, there is no research on humans, and its specific mechanism is still unclear. We found that the gray matter volume of the left superior frontal gyrus, right superior parietal lobule, and cerebellum in idiopathic tinnitus patients with mild hearing loss was significantly higher than that in tinnitus patients with normal hearing. However, due to the baseline mismatch between the two groups, it is not considered that mild hearing loss will lead to gray matter degradation in these areas. Interestingly, Koops et al. compared the changes in gray matter volume in hearing loss patients with and without tinnitus and found that both the auditory-related cortex and nonauditory cortex of hearing loss patients without tinnitus showed a decrease in gray matter volume, while this phenomenon was not observed in hearing loss patients with tinnitus [39]. This seems to contradict our results and is counterintuitive. This may be because the subjects included by Koops et al. who shared tinnitus and hearing loss had more severe hearing loss compared to this study, mainly manifested as difficulties related to hearing loss, and tinnitus was not their primary complaint. The interaction between hearing loss and tinnitus in neuroimaging still needs further research.

This study found differences in multiple neuroanatomical parameters of the parahippocampal gyrus in patients with idiopathic tinnitus, which may suggest that the parahippocampal gyrus, as an important component of the limbic system [40], plays a crucial role in the central plasticity changes of tinnitus patients. In addition, we also found that the SAS and SDS scores of patients with idiopathic tinnitus were significantly positively correlated with the cortical thickness of the bilateral parahippocampal gyrus and the gyrification index of the left parahippocampal gyrus. This provides strong evidence for explaining the role of the parahippocampal gyrus in emotional-related symptoms in patients with idiopathic tinnitus. The neural structural changes in this region are influenced by multiple factors, emphasizing that tinnitus patients may be experiencing similar psychological distress as psychiatric patients. Besteher et al. [32] and Wei et al. [41] also found a decrease in tinnitus patients’ gray matter volume in the parahippocampal gyrus. In addition, Schmidt et al. emphasized that the gray matter volume of the left parahippocampal gyrus was reduced in patients with severe tinnitus compared to those with mild tinnitus [31], once again confirming the close correlation between the parahippocampal gyrus and the comorbid symptoms of tinnitus. Interestingly, Sun et al. recently discovered that a larger gray matter volume in the posterior left parahippocampal gyrus may increase the risk of tinnitus [42]. The important role of the parahippocampal gyrus in the occurrence and severity of tinnitus suggests that it may become a potential target for central targeted therapy of tinnitus. We also found similar manifestations in the insula, indicating that the emotional and psychological problems of tinnitus patients are the result of the joint action of multiple brain regions.

As the main mediator of cortical information entering and exiting the hippocampus [43], the entorhinal cortex exhibits distinctive morphological changes in patients with idiopathic tinnitus. The thickness of the bilateral entorhinal cortex in THL was significantly reduced compared to the control group, while the sulcal depth of the left entorhinal cortex decreased, and the gyrification index was significantly increased. However, there was no significant change observed in TNH. Yoo et al. found that the cortical thickness of the entorhinal cortex is negatively correlated with hearing loss and age [44], which is consistent with our results. The entorhinal cortex is believed to be involved in memory processing and information integration, especially during sleep [45]. However, it is worth considering that the structural characteristics of the entorhinal cortex do not show a significant correlation with PQSI and MoCA scores that refer to sleep and cognition, but their cortical thickness is positively correlated with SAS and SDS scores. Perhaps, the entorhinal cortex plays a different role than the normal structure in the central plasticity of tinnitus patients. Similarly, the fusiform, as a brain region highly associated with sleep, also exhibits a significant correlation with SAS and SDS scores in tinnitus patients. In addition, we also found structural changes in the cortex related to motor and executive functions, including the precentral gyrus and middle temporal gyrus, in tinnitus patients. These cortices are believed to be related to cognitive function [46]. Consistent with this, we found that the cortical thickness of the bilateral precentral gyrus, the cortical thickness of the right middle temporal gyrus, and the fractal dimension of the left middle temporal gyrus were positively correlated with the MoCA score.

Nicholas et al. pointed out that the high heterogeneity of subjects included in different studies particularly affects the reliability of exploring the etiology of tinnitus through structural MRI [15]. Thomas et al. conducted a small sample (n < 20) case-control matching to control for heterogeneity in tinnitus patients in a single analysis [14]. This study specifically grouped patients with idiopathic tinnitus based on their hearing level to explore the structural abnormalities in the brain between those with normal hearing and those with mild hearing loss. By setting strict inclusion and exclusion criteria, including limiting tinnitus as the primary complaint and excluding subjects with severe mental health issues and patients with any other history of otolaryngological or neurological disorders, we have to some extent reduced the heterogeneity of idiopathic tinnitus in clinical features in this study. This study may provide a template for subsequent neuroimaging changes in tinnitus, further standardizing research methods in this field.

However, there are still some shortcomings in this study. Although sufficient participants were included in this study, the sample size of tinnitus patients in both groups was still relatively small compared to the control group when performing subgroup analysis. Further research is needed to expand the sample size to meet the subgroup analysis of idiopathic tinnitus. We lack some longitudinal observations, which may have some significance in understanding neurostructural signatures of idiopathic tinnitus in the different stages or before and after treatment. Unfortunately, longitudinal studies on idiopathic tinnitus are difficult to perform, with high dropout rates and a lack of effective treatment options limiting the development of these studies. In addition to hearing loss, other comorbidities of tinnitus should also be considered as causes of central anatomical changes in patients with idiopathic tinnitus, such as sleep disorders, severe psychological problems (including suicidal ideation and attempted suicide), and cognitive dysfunction. Limitation of space forbids full treatment of the subject. In addition, research on individual-specific networks may be an effective tool for addressing the high heterogeneity of idiopathic tinnitus. This will be the focus of our further work.

## 5. Conclusions

In summary, there are extensive neuroanatomical alterations in tinnitus patients, and the changes in brain microstructure are mainly presented as the reduction of gray matter volume and the distinct pattern of surface parameters. Mild hearing loss may aggravate the reduction of gray matter volume and change the surface characteristics of the cortex. In addition, we also found that anxiety, depression, and cognitive impairment in patients with idiopathic tinnitus may be related to neuroanatomical alterations in specific brain regions. This provides strong evidence for changes in the central plasticity of tinnitus and may represent a new target for tinnitus treatment.

## Figures and Tables

**Table 1:** Demographic and clinical characteristics.

	THL (n = 31)	TNH (n = 43)	HC (n = 98)	Statistics	*p*
Age (years)	52.6 ± 10.6	44.7 ± 11.4	43.4 ± 13.1	F = 6.81	0.001
Gender (male/female)	15/16	18/25	50/48	χ^2^ = 1.01	0.605
Education (years)	12.8 (9.5, 15.7)	13.0 (10.0, 15.8)	12.9 (10.2, 15.4)	H = 0.10	0.951
PTA of better ear	28.1 ± 7.9	13.1 ± 4.5	11.2 ± 8.6	F = 57.82	<0.001
PTA of worse ear	32.7 ± 9.3	17.8 ± 6.9	14.4 ± 9.4	F = 45.45	<0.001
MoCA scores	21.4 ± 4.0	23.4 ± 3.8	27.1 ± 1.9	F = 44.38	<0.001
SAS scores	28.0 (25.7, 31.0)	29.3 (26.9, 35.1)	24.4 (21.5, 29.5)	H = 17.02	<0.001
SDS scores	27.9 (25.5, 33.1)	30.0 (26.9, 35.9)	28.0 (23.3, 33.3)	H = 5.39	0.068
PQSI scores	9.5 ± 4.2	9.1 ± 3.6	5.2 ± 2.9	F = 26.09	<0.001
Sides (left/right/bilateral)	7/8/16	9/10/24		χ^2^ = 0.13	0.937
Duration (months)	13.5 (10.1, 34.5)	19.1 (10.3, 49.0)		T = 0.28	0.777
THI scores	24.9 ± 15.9	27.6 ± 17.4		t = 0.68	0.501
Tf (low/mid/high)	4/12/15	9/14/20		χ^2^ = 0.87	0.648
Tinnitus loudness	57.1 ± 15.0	35.5 ± 12.7		t = 6.67	<0.001

Note: THL, idiopathic tinnitus patients with mild hearing loss; TNH: idiopathic tinnitus patients with normal hearing; HC: healthy control; PTA: Mean pure tone thresholds at 500, 1000, 2000, and 4000 Hz; MoCA: Montreal Cognitive Assessment; SAS: Self-Rating Anxiety Scale; SDS: Self-Rating Depression Scale; PQSI: Pittsburgh Sleep Quality Index; THI: Tinnitus Handicap Inventory; Tf: tinnitus frequency, low: ≤1000 Hz, mid: 1001–4000 Hz, high: >4000 Hz.

**Table 2:** Gray matter volume differences among three groups (uncorrected *p* < 0.001).

Contrast	Side	Brain Regions	Cluster	MNI (X, Y, Z)			T Max
HC > THL	L	Cerebrum, Frontal lobe, Middle frontal gyrus	754	−42	23	54	4.41
		Cerebrum, Frontal lobe, Superior frontal gyrus	407	−10	−14	69	4.09
		Limbic lobe, Cerebrum, Cingulate gyrus, Cingulum	251	−3	5	27	4.09
		Cerebrum, Cingulum, Limbic lobe, Anterior cingulate	170	−7	45	13.5	3.41
		Cerebrum, Postcentral gyrus, Parietal lobe	169	−39	26	50	3.79
		Cerebrum, Frontal lobe, Superior frontal gyrus,	168	−18	63	29	3.55
		Frontal lobe, Cerebrum, Inferior frontal gyrus	130	−57	14	30	3.90
		Cerebrum, Parietal lobe, Precuneus	111	−30	−72	39	3.76
		Midbrain, Left brainstem, Right brainstem	84	−3	−30	−6	3.56
	R	Cerebrum, Frontal lobe, Precentral gyrus	462	39	−18	66	3.81
		Cerebrum, Temporal lobe, Superior temporal gyrus, Inferior frontal gyrus	221	42	14	−15	3.58
		Temporal lobe, Right cerebrum, Temporal pole, Middle temporal gyrus	147	48	8	−33	3.50
		Parietal lobe, Cerebrum	125	21	−59	57	3.67
HC > TNH	L	Frontal lobe, Cerebrum, Precentral gyrus	335	−42	−9	68	3.99
		Cerebrum, Parietal lobe, Postcentral gyrus	31	−21	−42	63	3.29
	R	Cerebrum, Temporal lobe	37	38	−4	−32	3.45
TNH > THL	L	Frontal lobe, Cerebrum, Superior frontal gyrus	69	−15	59	20	3.68
	R	Parietal lobe, Cerebrum, Superior parietal lobe	161	31	−60	59	4.16

Note: MNI: Montreal Neurological Institute coordinates; THL, idiopathic tinnitus patients with mild hearing loss; TNH: idiopathic tinnitus patients with normal hearing; HC: healthy control.

**Table 3:** Cortical thickness differences among three groups (uncorrected *p* < 0.001).

Contrast	Side	Brain Regions	Cluster	MNI (X, Y, Z)			T Max
HC > THL	L	Parahippocampal gyrus, Entorhinal cortex	327	−22	−3	−35	4.49
		Precentral gyrus	127	−49	−11	46	3.67
		Lateral orbitofrontal cortex, Insula	53	−27	20	−11	3.92
	R	Precentral gyrus	398	51	−2	49	4.84
		Parahippocampal gyrus	167	29	−30	−21	4.06
		Fusiform, Entorhinal cortex	53	30	−7	−35	3.61
TNH > HC	L	Fusiform, Lingual gyrus	114	−28	−58	−13	4.10
		Supramarginal gyrus	99	−43	−30	19	4.22
		Superior frontal gyrus, Rostral middle frontal cortex	91	−18	50	35	3.74
		Peri calcarine, cuneus	81	−7	−84	10	3.61
		Lingual gyrus	29	−6	−65	3	3.42
	R	Inferior parietal lobe	84	49	−52	44	3.66
		Superior frontal gyrus	46	20	34	45	3.44
		Fusiform	44	34	−49	−19	3.74
		Rostral middle frontal cortex	35	28	47	16	3.42
TNH > THL	L	Supramarginal gyrus, Superior temporal gyrus	70	−38	−35	18	3.78
		Superior temporal gyrus, insula	51	−43	−21	−6	3.48
	R	Inferior parietal lobe	138	47	−58	27	4.30
		Middle temporal gyrus, Bankssts, Inferior parietal lobe	117	57	−58	7	4.24
		Precentral gyrus, caudal middle frontal cortex	109	45	3	46	4.29
		Fusiform, Entorhinal cortex	99	31	−5	−37	4.23
		Inferior parietal lobe	56	44	−50	42	3.78
		Inferior parietal lobe	33	45	−69	10	3.55

Note: MNI: Montreal Neurological Institute coordinates; THL, idiopathic tinnitus patients with mild hearing loss; TNH: idiopathic tinnitus patients with normal hearing; HC: healthy control.

**Table 4:** Cortical sulcal depth differences among three groups (uncorrected *p* < 0.001).

Contrast	Side	Brain Regions	Cluster	MNI (X, Y, Z)			T Max
HC > TNH	L	Precentral gyrus	17	−55	5	1	3.37
		Rostral middle frontal cortex	16	−38	54	−2	3.42
HC > THL	L	Postcentral gyrus, Precentral gyrus	102	−34	−29	52	3.49
		Fusiform, Entorhinal cortex	56	−34	−10	−36	3.75
		Superior frontal gyrus	22	−8	54	16	3.33
		Rostral anterior cingulate, Medial orbitofrontal cortex	21	−11	43	−3	3.27
	R	Precentral gyrus, Caudal middle frontal cortex	156	41	−13	63	3.88
TNH > THL	R	Superior temporal gyrus	27	52	7	−12	3.56

Note: MNI: Montreal Neurological Institute coordinates; THL, idiopathic tinnitus patients with mild hearing loss; TNH: idiopathic tinnitus patients with normal hearing; HC: healthy control.

**Table 5:** Cortical gyrification index differences among three groups (uncorrected *p* < 0.001).

Contrast	Side	Brain Regions	Cluster	MNI (X, Y, Z)			T Max
HC > TNH	L	Superior temporal gyrus, Supramarginal gyrus	57	−60	−53	16	3.66
		Cuneus, Precuneus, Peri calcarine	43	−21	−64	16	3.43
		Superior temporal gyrus	36	−47	−36	10	3.25
	R	Middle temporal gyrus	26	58	−52	−4	3.41
THL > HC	L	Temporal pole, Entorhinal cortex	147	−23	4	−38	4.67
THL > TNH	L	Entorhinal cortex, Temporal pole, Fusiform	38	−24	3	−41	3.67
		Superior temporal gyrus	21	−55	8	−13	3.58
		Entorhinal cortex, Parahippocampal gyrus	12	−23	−18	−24	3.84

Note: MNI: Montreal Neurological Institute coordinates; THL, idiopathic tinnitus patients with mild hearing loss; TNH: idiopathic tinnitus patients with normal hearing; HC: healthy control.

**Table 6:** Cortical fractal dimension differences among three groups (uncorrected *p* < 0.001).

Contrast	Side	Brain Regions	Cluster	MNI (X, Y, Z)			T Max
HC > THL	L	Lateral orbitofrontal cortex, Insula	82	−30	18	−19	4.00
		Post central gyrus	38	−63	−13	21	3.46
		Middle temporal gyrus	25	−59	−8	−21	3.39
	R	Parahippocampal gyrus, Fusiform	110	36	−37	−13	3.90
		Precuneus, Cuneus	20	21	−70	22	3.47
		Superior parietal lobe	15	17	−53	60	3.31
THL > HC	L	Lateral occipital cortex	82	−33	−82	0	3.95
HC > TNH	L	Medial orbitofrontal cortex	15	−4	44	−26	3.31
	R	Superior temporal gyrus, Temporal pole	39	40	7	−25	3.73
TNH > THL	R	Superior parietal lobe	100	18	−53	60	4.08
		Fusiform, Parahippocampal gyrus	55	37	−38	−14	3.63
THL > TNH	L	Lateral occipital cortex	84	−29	−85	2	4.22
	R	Inferior temporal gyrus	12	46	−18	−30	3.46

Note: MNI: Montreal Neurological Institute coordinates; THL, idiopathic tinnitus patients with mild hearing loss; TNH: idiopathic tinnitus patients with normal hearing; HC: healthy control.

**Table 7:** Correlation between clinical and neuroimaging characteristics (*p* < 0.01).

Clinical Characteristics	Neuroimaging Characteristics	Brain Regions	R	*p*
SAS scores	Cortical thickness	Left Entorhinal cortex	0.58	<0.001
		Right Entorhinal cortex	0.56	<0.001
		Left Fusiform	0.38	<0.001
		Right Fusiform	0.34	0.003
		Left Parahippocampal gyrus	0.59	<0.001
		Right Parahippocampal gyrus	0.55	<0.001
		Left insula	0.38	<0.001
		Right insula	0.38	<0.001
	Cortical gyrification index	Left Parahippocampal gyrus	−0.35	0.003
	Cortical fractal dimension	Left Lateral orbitofrontal cortex	0.36	0.002
SDS scores	Cortical thickness	Left Entorhinal cortex	0.53	<0.001
		Right Entorhinal cortex	0.50	<0.001
		Left Fusiform	0.42	<0.001
		Left Parahippocampal gyrus	0.52	<0.001
		Right Parahippocampal gyrus	0.49	<0.001
		Left insula	0.38	<0.001
		Right insula	0.34	0.003
	Cortical gyrification index	Left Parahippocampal gyrus	−0.34	0.003
	Cortical fractal dimension	Left Lateral orbitofrontal cortex	0.34	0.003
MoCA scores	Cortical thickness	Right Inferior parietal lobe	0.31	0.007
		Left Lingual gyrus	0.42	<0.001
		Right Middle temporal gyrus	0.30	<0.008
		Left Precentral gyrus	0.32	0.006
		Right Precentral gyrus	0.32	0.006
		Right Superior temporal gyrus	0.31	0.008
	Cortical fractal dimension	Left Middle temporal gyrus	0.36	0.002

## Data Availability

The original contributions presented in the study are included in the article; further inquiries can be directed to the corresponding authors.
